# Diffusion Tensor Imaging Along the Perivascular Space Index in Different Stages of Parkinson’s Disease

**DOI:** 10.3389/fnagi.2021.773951

**Published:** 2021-11-15

**Authors:** Xinxin Ma, Shuhua Li, Chunmei Li, Rui Wang, Min Chen, Haibo Chen, Wen Su

**Affiliations:** ^1^Department of Neurology, Parkinson’s Disease and Extra Pyramidal Disease Diagnosis and Treatment Center, Beijing Hospital, National Center of Gerontology, Institute of Geriatric Medicine, Chinese Academy of Medical Sciences, Beijing, China; ^2^Department of Radiology, Beijing Hospital, National Center of Gerontology, Institute of Geriatric Medicine, Chinese Academy of Medical Sciences, Beijing, China

**Keywords:** Parkinson’s disease, glymphatic system, magnetic resonance imaging, diffusion tensor imaging, enlarged perivascular spaces

## Abstract

**Background:** The aim of this study was to evaluate the glymphatic system activity in patients with Parkinson’s disease (PD) using the diffusion tensor image analysis along the perivascular space (DTI-ALPS) methods.

**Methods:** In total, 71 patients with idiopathic PD and 36 age- and sex-matched normal controls (NCs) were involved. Patients with PD were divided into early (*n* = 35) and late (*n* = 36) subgroups, based on Hoehn and Yahr (HY) stages. We calculated the diffusivity along the perivascular spaces (ALPS), as well as projection fibers and association fibers separately, to acquire the ALPS index. Enlarged perivascular spaces (EPVS) and periventricular white matter hyperintensities were also rated. Differences in ALPS index between the PD group and NCs and between two PD subgroups and NCs were compared. In addition, a multivariate logistic regression analysis was conducted to investigate the association between ALPS index and clinical variables.

**Results:** Patients with PD revealed lower ALPS index than NCs (*p* = 0.010). The late PD group exhibited significantly lower ALPS index than NCs (*p* = 0.006). However, there were no marked differences noticed in ALPS index between NCs and early PD group and between the two PD subgroups. In the early PD group, there was a significantly positive correlation between ALPS index and Mini-Mental State Examination (MMSE) score (β = 0.021, *p* = 0.029) and a negative correlation between ALPS index and EPVS score (β = −0.050, *p* = 0.034), after controlling for multiple variables. In the late PD group, ALPS index was inversely associated with age (β = −0.012, *p* = 0.004).

**Conclusion:** Impairment of the glymphatic system is involved in PD. DTI-ALPS index could be a promising biomarker of glymphatic system in PD.

## Introduction

Parkinson’s disease (PD) is a common neurodegenerative disease, but the underlying mechanism is poorly understood. In addition to the loss of dopaminergic neurons, the aggregation of misfolded α-synuclein (α-syn) is also involved in the pathogenesis of PD (George et al., 2013; Poewe et al., 2017). The glymphatic system was first identified by Iliff et al. (2012), which was defined as a waste excretion system in the brain. Cerebrospinal fluid (CSF) is exchanged with interstitial fluid along the perivascular space (PVS) to promote the elimination of soluble proteins, including misfolding proteins and metabolites (Iliff et al., 2012; Benveniste et al., 2019). Therefore, this system has been thought to play a critical role in the pathophysiology of PD.

Recently, Taoka et al. (2017) proposed a non-invasive method “diffusion tensor image analysis along the perivascular space” (DTI-ALPS) to assess the glymphatic function in human brain. They indicated that ALPS index positively correlated with the Mini-Mental State Examination (MMSE) score in Alzheimer’s disease (AD; Taoka et al., 2017). Subsequently, this method has been used in patients with dementia (Steward et al., 2021), idiopathic normal pressure hydrocephalus (iNPH; Yokota et al., 2019; Bae et al., 2021), and type 2 diabetes mellitus (Yang et al., 2020). To our knowledge, there are only two studies exploring the glymphatic system activity in patients with PD, using DTI-ALPS method (Chen et al., 2021; McKnight et al., 2021). They highlighted that patients with PD with cognitive impairment showed lower ALPS index than normal controls (NCs). Moreover, patients with PD exhibited reduced ALPS index related to patients with essential tremor. However, the association between ALPS index and various clinical factors in different stages of PD has not yet been reported. Therefore, we aimed to investigate the glymphatic function in patients with PD, using this non-invasive method. We also examined the related factors of the ALPS index in different stages of PD.

In addition, several lines of evidence have emerged suggesting that PVS are associated with the clearance of interstitial fluid and waste from the brain. Enlarged perivascular spaces (EPVS) on MRI may reflect impairment of lymphatic drainage channels (Wardlaw et al., 2020). Lower grade of EPVS predicted lower CSF α-syn and t-tau in PD (Fang et al., 2020). However, it has not been established in humans as to whether MRI-visible EPVS are related to DTI ALPS index in PD. Hence, we also explored the relationship between EPVS and ALPS index in different stages of PD. This study may help us to unravel the glymphatic system activity in PD and find potential neuroimaging markers for diagnosing and monitoring the progression of PD.

## Materials and Methods

### Subjects and Clinical Assessments

Patients with idiopathic PD (*n* = 71, mean age: 64.68 ± 8.12 years) and age- and sex-matched NCs (*n* = 36, mean age: 62.00 ± 6.24 years) were involved in this study. All patients with PD were diagnosed based on the United Kingdom PD Society Brain Bank Clinical Diagnostic Criteria. All participants were right-handed Chinese natives. We excluded patients in whom PD was induced by cerebrovascular disease, medications, trauma, encephalitis, poisoning, and other neurodegenerative diseases. Neurological examinations were evaluated using MMSE score, Unified Parkinson’s Disease Rating Scale (UPDRS) score, and Hoehn and Yahr (HY) stages. Patients with PD receiving dopaminergic medications were examined in the clinically defined “OFF” state. All neuropsychological scales were completed by one neurologist who was blinded to the clinical information. In addition, patients were divided into early PD group (HY 1–2, *n* = 35) and late PD group (HY 2.5–4, *n* = 36), based on HY stages. This study was approved by a local ethics committee, and written informed consent was obtained from each participant after a detailed description of the study was provided.

### MR Image Acquisition

All MRI examinations were performed using a 3.0 T MRI scanner (Philips, Achieva TX). The head of the participant was immobilized with foam pillows inside the coil to diminish motion artifacts. Sequences consisted of high-resolution T1-weighted 3D (repetition time/echo time (TR/TE) = 7.4/3 ms, flip angle (FA) = 8, field of view (FOV) = 24 cm × 24 cm, and 1.2 mm slice thickness without slice gap), T2-weighted (T2WI; TR/TE = 2,500/100 ms; FOV = 24 cm × 24 cm, 5 mm slice thickness, and 1.5 mm slice gap), fluid-attenuated inversion recovery (FLAIR; TR/TE = 8,000/140 ms; TI = 2,400 ms; FOV = 24 cm × 24 cm, and 4 mm slice thickness without slice gap), susceptibility weighted imaging (SWI; TR/TE = 16/22 ms; FOV = 24 cm × 24 cm; and 2.8 mm slice thickness without slice gap), and diffusion tensor imaging (axially parallel to the anterior-posterior commissure (AC-PC), TR/TE = 5,472/93 ms; b = 1,000 s/mm^2^; FOV = 24 cm × 24 cm; matrix = 128 × 128; MPG = 31 directions; 3 mm slice thickness without slice gap, 40 slices).

### MRI Analysis

**FIGURE 1:**
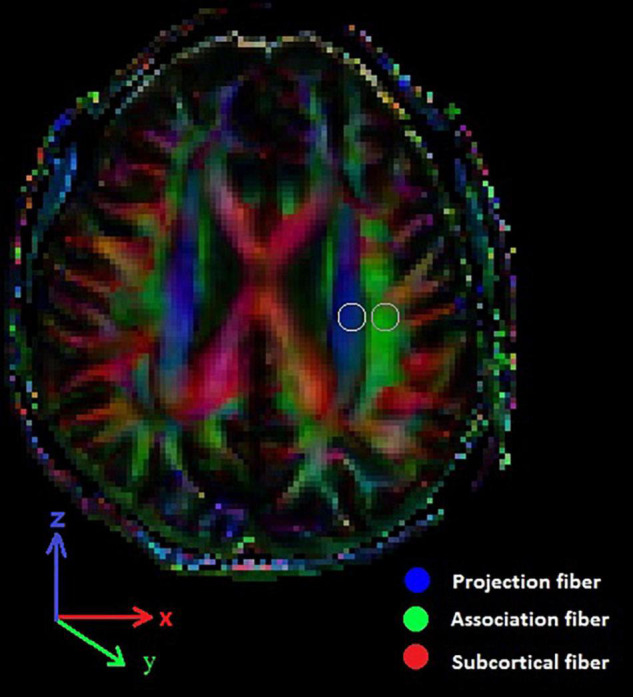
DTI color map shows the direction of the projection fibers (blue; z-axis), association fibers (green; y-axis), and the subcortical fibers (red; x-axis). Two ROIs are placed to measure diffusivities of the projection and association fibers.

The DTI data were calculated using DTI Studio software.^1^ The software creates images of the diffusion tensor, including a color-coded fractional anisotropy (FA) map and a diffusivity map. Moreover, the diffusivity in the directions of *x*-axis, *y*-axis, and *z*-axis on each image can be calculated. We evaluated the diffusivity along the direction of the PVS compared with those of projection fibers and association fibers on a slice at the level of the lateral ventricle body (Figure 1). At this level, the direction of the PVS is perpendicular to the ventricle wall (mostly in the right-left direction/*x*-axis). This direction is also perpendicular to the direction of the projection fibers (mostly in the *z*-axis) as well as the association fibers (mostly in the *y*-axis) (Figure 1). Therefore, the diffusivity along the *x*-axis at regions with projection/association fibers will at least partly represent the diffusivity along the PVS. On a color-coded FA map, we placed a 5-mm-diameter spherical region of interest (ROI) in the area of the projection fibers (represented in blue in Figure 1) and the area of the association fibers (represented in green in Figure 1) in the left hemisphere. For each area, the diffusivity in the directions of the *x*-axis, *y*-axis, and *z*-axis was calculated.

We also calculated the ALPS index to assess the activity of the glymphatic system. According to a study by Taoka et al. (2017), this index is provided by the ratio of two diffusivity value sets, i.e., the ratio of the average values of the *x*-axis diffusivity in the area of the projection fibers (D_*x*__*xproj*_) and the *x*-axis diffusivity in the area of the association fibers (D_*x*__*xassoc*_) to the average value of the *y*-axis diffusivity in the area of the projection fibers (D_*y*__*yproj*_) and the *z*-axis diffusivity (D_*z*__*zacoc*_) of the association fibers area, shown as follows:

ALPS index = mean (D_*x*__*x*__*proj*_, D_*x*__*x*__*assoc*_)/mean (D_*y*__*y*__*proj*_, D_*z*__*z*__*assoc*_). The ALPS index represents the existence of the PVS. A higher ratio represents the more water diffusivity along the PVS.

In addition, PVSs were defined as punctate hyperintensities on T2WI at the same level of the lateral ventricle body, usually<3 mm in diameter, based on a previous work (Doubal et al., 2010). Periventricular white matter hyperintensities (PVWMH) were also investigated using the Fazekas scale from 0 to 3 (Fazekas et al., 1987). All MRI lesions were assessed by two trained neurologists who were blinded to the clinical information of participants.

### Statistical Analysis

Statistical Package for the Social Sciences (SPSS) 25.0 software was used to perform the statistical analysis of clinical and demographic variables. Two-sample *t*-test and chi-square test were conducted to examine the clinical differences of continuous variables and categorical variables, respectively, between PD and NCs, and between ePD and lPD groups. In addition, analysis of variance (ANOVA) and *post hoc* tests were used to compare the differences between NCs and two PD subgroups. Multivariate linear regression analysis was carried out between the ALPS index and clinical variables in the early and late PD groups, respectively, including age, sex ratio (the ratio of males to females in a population), duration, MMSE score, EPVS score, UPDRS part III and total score, and HY stages. *p* < 0.05 was considered statistically significant.

## Results

**FIGURE 2:**
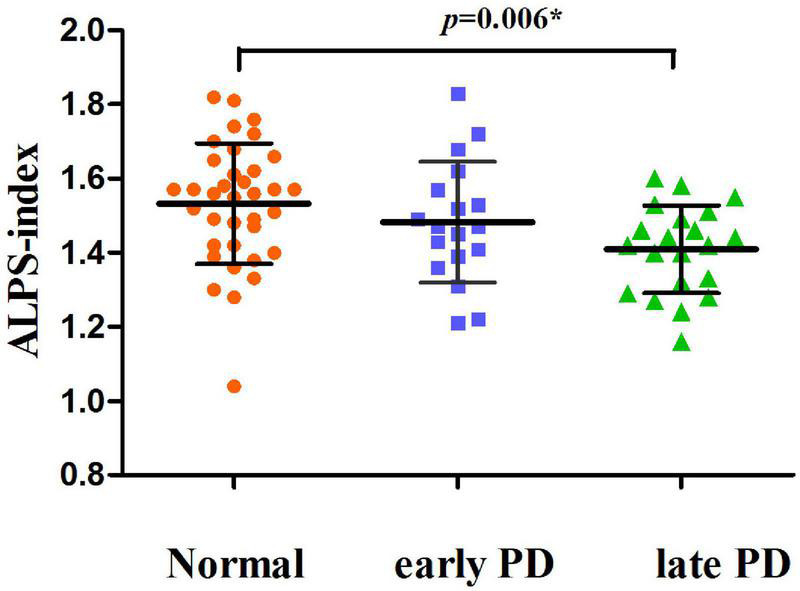
Scatter plots showing the distribution of the ALPS-index values for normal controls, early and late PD subgroups. The *p*-value was obtained from the analysis of variance (ANOVA) *post hoc* tests.

We involved 71 patients with PD and 36 NCs in this study. There were no significant differences in age and sex ratio between the PD and NC groups. Demographic and clinical data of the subjects are presented in Table 1. Patients with PD revealed reduced ALPS index than NCs (*p* = 0.010). The late PD group exhibited significantly lower ALPS index than NCs (*p* = 0.006). However, there were no marked differences in ALPS index between the NCs and the early PD group, and between the two PD subgroups (Figure 2). These three groups did not differ in age, sex ratio, and MMSE score (*p* > 0.05). While two PD subgroups showed higher education than NCs. Compared with the early PD group, the late PD group revealed longer disease duration, higher UPDRS part II, part III, total score, and higher PDQ-39 score (Table 1). No significant difference in ALPS index and EPVS score between the two PD groups was observed.

**TABLE 1:** Demographic and clinical data of the subjects.

	**NCs (*n* = 36)**	**PD (*n* = 71)**	***p***	**Early PD (*n* = 35)**	**Late PD (*n* = 36)**	***p***
Age (years)	62.00 ± 6.24	64.68 ± 8.12	0.062	63.57 ± 8.93	65.75 ± 7.23	0.110
Sex (M/F)	18/18	31/40	0.534	17/18	14/22	0.590
MMSE	27.86 ± 2.20	28.03 ± 2.15	0.707	28.09 ± 2.47	27.97 ± 1.83	0.910
EDU (years)	10.44 ± 3.18	12.93 ± 3.30	0.000^*a*^	12.91 ± 3.48	12.94 ± 3.16	0.002^b^
ALPS index	1.53 ± 0.16	1.44 ± 0.16	0.010^*a*^	1.46 ± 0.15	1.42 ± 0.18	0.022^b^
Dur (years)	NA	8.38 ± 4.29	NA	6.86 ± 4.02	9.86 ± 4.06	0.002^b^
UPDRS-I	NA	3.04 ± 1.93	NA	2.80 ± 2.13	3.28 ± 1.72	0.300
UPDRS-II	NA	12.79 ± 4.93	NA	10.40 ± 4.31	15.11 ± 4.4	0.000^b^
UPDRS-III	NA	30.92 ± 11.58	NA	26.31 ± 9.80	35.39 ± 11.54	0.001^b^
UPDRS-IV	NA	2.68 ± 2.46	NA	2.14 ± 2.35	3.19 ± 2.49	0.072
UPDRS-V	NA	1.06 ± 0.95	NA	0.97 ± 1.04	1.14 ± 0.87	0.464
UPDRS-total	NA	50.48 ± 17.25	NA	42.63 ± 14.76	58.11 ± 16.19	0.000^b^
EPVS	NA	1 (0–4)	NA	1 (0–4)	1 (0–4)	0.663
PVWMH	NA	2 (0–3)	NA	1 (0–3)	2 (1–3)	0.130

*M/F, male/female; NCs, normal controls; PD, Parkinson’s disease; MMSE, Mini-Mental State Examination; EDU, education; ALPS, along the perivascular spaces; Dur, disease duration; UPDRS, Unified Parkinson’s Disease Rating Scale; EPVS, enlarged perivascular spaces; PVWMH, periventricular white matter hyperintensities.*

*^a^Two-sample t-test between NCs and PD.*

*^b^Two-sample t-test between early and late PD groups.*

*p < 0.05.*

**FIGURE 3:**
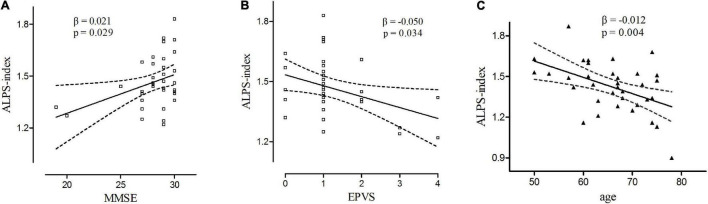
**(A)** Relationships between ALPS-index and MMSE score in early PD group. **(B)** Relationships between ALPS-index and EPVS score in early PD group. **(C)** Relationships between ALPS-index and age in late PD group. Trend lines describe multivariate linear regression with 95% confidence intervals (dashed line).

In multivariate linear regression analysis, there was a significant positive correlation between ALPS index and MMSE score (β = 0.021, *p* = 0.029) (Figure 3A), and a negative correlation between ALPS index and EPVS score (β = −0.050, *p* = 0.034) (Figure 3B) in the early PD group. However, ALPS index did not associate with age, sex ratio, duration, UPDRS-III, UPDRS total score, HY stages, and PVWMH score in this group. In the late PD group, ALPS index negatively related with age (β = −0.012, *p* = 0.004) (Figure 3C). However, there was no significant relationship between ALPS index and other clinical status in this group (*p* > 0.05).

After controlling for sex ratio, disease duration, MMSE score, HY stages, UPDRS part III and total score, and PVWMH score, EPVS score was positively related to age in all PD participants (β = 0.056, *p* = 0.000), and in the early (β = 0.059, *p* = 0.000) and late PD (β = 0.053, *p* = 0.023) subgroups, respectively.

## Discussion

This study suggested that patients with PD exhibited lower ALPS index than NCs, especially in the late stage. ALPS index positively correlated with cognitive function in the early stage of PD and was inversely related to age in the advanced stage. Furthermore, we first demonstrated a negative association between the ALPS index and EPVS score in the early PD group. Our findings provided evidence supporting the involvement of glymphatic system in the pathogenesis of PD.

The glymphatic-lymphatic system is a recently discovered brain-wide paravascular pathway for the CSF and interstitial fluid exchange, which promotes efficient clearance of soluble proteins and metabolites from the brain (Iliff et al., 2013). Reduced clearance of protein waste from the brain has been observed in several neurological disorders (Rasmussen et al., 2018). However, the studies supporting the glymphatic system dysfunction in PD are relatively limited.

Lewy pathology has been hypothesized to a hallmark of PD, which consists of abnormal aggregates of α-syn protein. A previous work has highlighted the involvement of α-syn in the pathogenesis of PD (George et al., 2013). There was an association between α-syn species concentrations in the CSF and blood and motor progression in patients with PD (Parnetti et al., 2019). Ding et al. (2021) also confirmed the impairment of meningeal lymphatic system in patients with PD compared with atypical parkinsonian disorders. Their findings revealed that meningeal lymphatic drainage dysfunction may aggravate α-syn pathology and exacerbate PD progression (Ding et al., 2021). Ineffective elimination of α-syn, which is closely related to the dysfunction of glymphatic system, may be a contributory factor to the development of PD. Most of the studies investigating the role of the glymphatic system were conducted in animal models. Finding the effective glymphatic system biomarkers in human is still challenging. Recently, Taoka et al. (2021) proposed a new non-invasive method DTI-ALPS to help assess the glymphatic system activity in human. They also suggested that ALPS index was robust under unified imaging method but was influenced by the imaging plane, the number of motion-proving gradient axes, and TE in the imaging sequence (Taoka et al., 2021). Until present, there are only two studies using DTI-ALPS method to evaluate glymphatic system activity in patients with PD (Chen et al., 2021; McKnight et al., 2021). Chen et al. (2021) indicated that cognitively impaired patients with PD showed lower ALPS index than NCs. Moreover, glymphatic system dysfunction was associated with increased oxidative stress status in PD (Chen et al., 2021). McKnight et al. (2021) recently demonstrated that in comparison with essential tremor (ET) patients, ALPS index was reduced in patients with PD. However, there was no significant relationship between ALPS index and mild cognitive impairment status after controlling for age, sex, diagnosis status (PD or ET), and Fazekas score (McKnight et al., 2021). Our findings provided evidence supporting the notion that dysfunction of the glymphatic system has been implicated in the pathogenesis of PD, especially in the late stage. However, there was no significant difference in ALPS index between the early and late PD groups. Future studies are needed to determine the utility of DTI-ALPS index in monitoring the progression of PD.

Furthermore, Taoka et al. proposed a new concept “central nervous system (CNS) interstitial fluidopathy” that represents diseases with impaired fluid dynamics of the CNS interstitial space (Taoka and Naganawa, 2021). These diseases include sleep disorders, AD, iNPH, traumatic brain injury, stroke, glaucoma, and other disorders. Our results support that PD has characteristics as a CNS interstitial fluidopathy. This term could also promote our understanding of the mechanisms and develop potential novel therapies in future.

A significant correlation between ALPS index and cognitive function has been observed in a previous study (Steward et al., 2021). Irwin et al. (2012) emphasized the importance of cortical Lewy bodies as pathological substrates of cognitive impairment and dementia in PD. Our result was consistent with previous studies. We first revealed that ALPS index was positively linked with MMSE score in the early stage of PD. While in the late stage, the correlation between ALPS index and age was noteworthy. Our findings provided insight into the role of glymphatic system on the cognitive function in PD. We also speculated that in the advanced stage of PD, increasing age may play a more vital role in the glymphatic system activity than other clinical factors.

In addition, our study first demonstrated a negative correlation between the ALPS index and EPVS score. EPVS on MRI has been considered as a marker of PVS dysfunction, which may reflect the impairment of normal brain fluid and waste clearance and microvascular dysfunction (Wardlaw et al., 2020). There is also a link between PVS in diffusion and structural scans. The influence of the PVS on the DTI-derived measures, including an increased mean diffusivity and decreased FA, has been demonstrated (Sepehrband et al., 2019). Previous work has indicated that the global and regional PVS volume fractions were increased in PD related to non-PD, especially in familial PD (FPD). A significant PVS volume fraction difference between idiopathic PD and FPD was also observed in the cuneus and lateral occipital regions (Donahue et al., 2021). Moreover, EPVS correlated with clinical symptoms in PD, such as tremor and cognitive decline (Park et al., 2019; Wan et al., 2019). However, a clinicopathological study found a lower prevalence of EPVS in PD group related to NCs (Schwartz et al., 2012). The interaction between EPVS and PD is still controversial. Recently, Li et al. (2020) revealed that patients with PD with EPVS in the substantia nigra (SN) showed greater expression of tau protein in CSF and a trend toward reduced DAT binding than those without SN-EPVS. Their results suggested an association between EPVS and SN degeneration in PD (Li et al., 2020). Another study also showed that patients with PD had a higher PVS burden in BG and midbrain than NCs, and the PVS number in BG significantly correlated with disease severity and L-dopa equivalent dosage (Shen et al., 2021). We speculated that a reduced glymphatic activity results in the accumulation of the misfolding proteins such as α-syn, which is implicated in the development of PVS dilation on MRI. Impaired clearance of large molecules may consequently stack in the PVSs, resulting in compensatory dilation.

There were some limitations in this study. (1) The sample size was relatively small. Larger sample size studies in this field are needed in future. (2) The DTI-ALPS method could calculate only the diffusivity along the direction of the PVS on one slice of the brain. We did not measure the diffusivity outside this area. (3) Head motion correction was not conducted in our study. But the head of the subject was immobilized with foam pillows inside the coil to diminish motion artifacts. Studies evaluating the optimal method are needed in future. (4) We could not use this method on an individual level. Further studies are warranted to develop new methods capable of differentiating patients on an individual level.

## Conclusion

Our findings revealed that patients with PD showed lower ALPS index than NCs, especially in the late stage of the disease. We demonstrated the dysfunction of glymphatic system in PD, which was correlated with cognitive impairment, EPVS on MRI, and increasing age. Furthermore, DTI-ALPS is a sensitive method to assess glymphatic system activity. Further studies are needed to determine the utility of DTI-ALPS index in diagnosing and monitoring the progression of PD.

## Data Availability Statement

The datasets presented in this article are not readily available because the original contributions presented in the study are included in the article/supplementary material, further inquiries can be directed to the corresponding author. Requests to access the datasets should be directed to WS, suwenbjyy@163.com.

## Ethics Statement

The studies involving human participants were reviewed and approved by the Beijing Hospital Ethics Committee. The patients/participants provided their written informed consent to participate in this study.

## Author Contributions

XM, HC, and WS contributed to the conception and design of the study. XM, SL, CL, RW, and MC organized the database and performed the statistical analysis. XM wrote the first draft of the manuscript. All authors approved the final version of the manuscript.

## Conflict of Interest

The authors declare that the research was conducted in the absence of any commercial or financial relationships that could be construed as a potential conflict of interest.

## Publisher’s Note

All claims expressed in this article are solely those of the authors and do not necessarily represent those of their affiliated organizations, or those of the publisher, the editors and the reviewers. Any product that may be evaluated in this article, or claim that may be made by its manufacturer, is not guaranteed or endorsed by the publisher.

## Footnotes

1
https://www.mristudio.org
